# Top–down assessment of the Asian carbon budget since the mid 1990s

**DOI:** 10.1038/ncomms10724

**Published:** 2016-02-25

**Authors:** R. L. Thompson, P. K. Patra, F. Chevallier, S. Maksyutov, R. M. Law, T. Ziehn, I. T. van der Laan-Luijkx, W. Peters, A. Ganshin, R. Zhuravlev, T. Maki, T. Nakamura, T. Shirai, M. Ishizawa, T. Saeki, T. Machida, B. Poulter, J. G. Canadell, P. Ciais

**Affiliations:** 1 Norsk Institutt for Luftforskning (NILU), Kjeller, Norway; 2 Department of Environmental Geochemical Cycle Research, Japan Agency for Marine Earth Science and Technology (JAMSTEC), Yokohama 236-0001, Japan; 3 Laboratoire des Sciences du Climat et l'Environnement (LSCE, CEA-CNRS-UVSQ), Gif-sur-Yvette, France; 4 National Institute for Environmental Studies (NIES), Tsukuba 305-8506, Japan; 5 Oceans and Atmosphere, Commonwealth Scientific and Industrial Research Organisation (CSIRO), 3195 Aspendale, Australia; 6 Department of Meteorology and Air Quality, Environmental Sciences Group, Wageningen University (WU), 6708 PB Wageningen, The Netherlands; 7 University of Groningen, Centre for Isotope Research, 9747 AG Groningen, The Netherlands; 8 Department of Upper Atmospheric Layers Physics, Central Aerological Observatory (CAO), Moscow 141700, Russia; 9 National Research Tomsk State University (TSU), 634050 Tomsk, Russia; 10 Department of Atmospheric Physics and Microwave Diagnostics, Institute of Applied Physics of the Russian Academy of Sciences, Nizhny Novgorod 603950, Russia; 11 Atmospheric Environment and Applied Meteorology Research Department, Meteorological Research Institute (MRI), Tsukuba 305-0052, Japan; 12 Japan Meteorological Agency (JMA), Global Environment and Marine Department, Tokyo 100-8122, Japan; 13 Institute on Ecosystems and Department of Ecology, Montana State University (MSU), 59717 Bozeman, Montana, USA; 14 Global Carbon Project, Commonwealth Scientific and Industrial Research Organisation (CSIRO), 2601 Canberra, Australia

## Abstract

Increasing atmospheric carbon dioxide (CO_2_) is the principal driver of anthropogenic climate change. Asia is an important region for the global carbon budget, with 4 of the world's 10 largest national emitters of CO_2_. Using an ensemble of seven atmospheric inverse systems, we estimated land biosphere fluxes (natural, land-use change and fires) based on atmospheric observations of CO_2_ concentration. The Asian land biosphere was a net sink of −0.46 (−0.70–0.24) PgC per year (median and range) for 1996–2012 and was mostly located in East Asia, while in South and Southeast Asia the land biosphere was close to carbon neutral. In East Asia, the annual CO_2_ sink increased between 1996–2001 and 2008–2012 by 0.56 (0.30–0.81) PgC, accounting for ∼35% of the increase in the global land biosphere sink. Uncertainty in the fossil fuel emissions contributes significantly (32%) to the uncertainty in land biosphere sink change.

## Abstract (web summary)

Land biosphere uptake of carbon is important in mitigating the anthropogenic increase in atmospheric CO_2_ and its climate forcing. Here, the authors show that land biosphere uptake of carbon in Asia has increased substantially since the mid 1990s, likely owing to reforestation and regional climate change.

## Graphical abstract

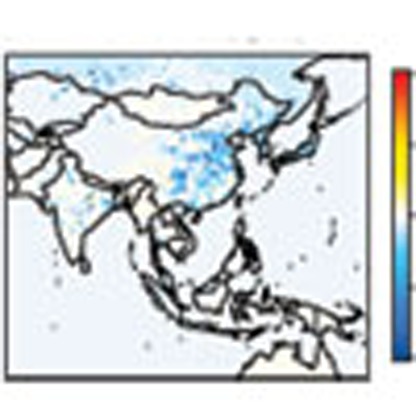

Increasing atmospheric carbon dioxide (CO_2_) is the single most important driver of climate change, and CO_2_ accounts for ∼64% of the radiative forcing from well-mixed greenhouse gases^1^. Hence reducing CO_2_ emissions is one of the principal concerns of efforts to mitigate anthropogenic climate change. The global mean atmospheric concentration of CO_2_ has increased from 277 parts-per-million (p.p.m.) in 1750 (ref. 2) to 396 p.p.m. in 2013 (ref. 3), due to emissions from fossil fuel combustion, cement production and land-use change. In the 1990s, the first international agreements to limit emissions of CO_2_ were ratified, the United Nations Framework Convention on Climate Change (UNFCCC), in 1992, and subsequently the Kyoto Protocol, in 1997. Very recently at the Conference of the Parties (COP21) meeting in Paris 2015, it was agreed that further efforts are needed to limit global warming to a maximum of 2 °C. To achieve this, peak greenhouse gas emissions should be reached as soon as possible and by the second half of this century anthropogenic emissions should be balanced by sinks (UNFCCC, CP21, L.9). For such agreements to be effectual, accurate means for assessing the effectiveness of regional and national emission reduction policies are critical, and require better estimates of CO_2_ emissions. Also, of upmost importance are improved estimates of the land and ocean biosphere sources and sinks and a better scientific understanding of the carbon cycle^4^.

Asia has undergone rapid economic growth over the past two decades, which has been associated with large increases in fossil fuel emissions. Fossil fuel emissions increased between 1990 and 2010 by ∼190% in India (to 0.55 PgC per year; 1 PgC = 1 × 10^15^ g carbon in CO_2_) and ∼240% in China (to 2.26 PgC per year) (Global Carbon Atlas, http://www.globalcarbonatlas.org) and in 2006 China surpassed the USA as the largest national emitter of CO_2_ (ref. 5). Asia has also seen substantial changes in land-cover over the same time period. Between 1990 and 2010, forest extent increased in South Asia, by ∼3% (2.1 Mha) and in East Asia by ∼22% (45.4 Mha), whereas in Southeast Asia deforestation occurred with a loss in forest extent of ∼13% (33.2 Mha)^6^. Deforestation rates are particularly extensive in Indonesia, where the rate of forest clearance (including forested peatlands) reached 0.84 Mha per year in 2012, surpassing that in Brazil^7^. These changes have important consequences for the regional and global carbon budget. Deforestation in Southeast Asia is a large source of CO_2_, releasing an estimated average of 0.23 PgC per year for 1990–2007 (ref. 8), while, East Asian ecosystems appear to be a substantial sink of CO_2_, taking up an estimated 0.16–0.33 PgC per year due to afforestation/reforestation and regional climate change, especially in Southern China^8^,^9^,^10^. The uncertainty associated with land-use change fluxes, however, is very large.

Top–down approaches, specifically atmospheric inversions, can help to reduce the uncertainty on land biosphere fluxes. Atmospheric inversions use observations of CO_2_ concentrations with a model of atmospheric transport to constrain CO_2_ fluxes between the land/ocean and atmosphere. Because the problem is under-determined, a prior estimate of the fluxes is needed, and this estimate is updated based on the atmospheric information to give a *posterior* estimate (see Methods). Although atmospheric inversions of CO_2_ have been used since the 1980s^11^,^12^, there have been few inversion studies focusing on Asia owing to the fact that until recently few atmospheric CO_2_ observations were available for this region. Recent programmes, such as CARIBIC (Civil Aircraft for the Regular Investigation of the atmosphere. On the basis of an Instrument Container)^13^, CONTRAIL (Comprehensive Observation Network for TRace gases by AIrLiner)^14^,^15^ and Asian Global Atmospheric Watch (GAW) have helped ameliorate this. CARIBIC data have been utilized in an inversion for CO_2_ fluxes in South Asia^16^ and CONTRAIL data in inversions focusing on Asian CO_2_ fluxes^17^,^18^,^19^. These inversions^16^,^17^,^18^ consistently found a net sink for South Asia of −0.11 to −0.37 PgC per year (note that a negative flux is in the direction from the atmosphere to the land/ocean, that is, a sink). In contrast, for Southeast Asia, one recent study^17^ found a source of ∼0.45 PgC per year, while another^18^ found a sink of ∼−0.28 PgC per year. Differences among inversion results can arise from differences in modelled atmospheric transport, prior information and its assumed uncertainty, as well as from the observations used. Therefore, to establish confidence in the magnitude and sign of regional CO_2_ fluxes, and to determine the range of uncertainty accounting not only for random errors, it is essential to examine ensembles of inversions.

In this study, we assess the carbon budget of Asia using an ensemble of seven atmospheric CO_2_ inversions focusing on the regions of East, South and Southeast Asia (see Fig. 1). The inversions use different prior information, transport models and observation data (see Supplementary Table 1) to estimate the land biosphere CO_2_ fluxes (including land-use change and fires) from 1996 to 2012. (Note that only four inversions are available for 1996 to 2000). All the inverse systems solve for the global CO_2_ fluxes with varying resolutions over the Asian regions. By using global inverse systems it is possible to infer the Asian fluxes consistently with the global carbon budget and the atmospheric growth rate of CO_2_ as well as assess how the optimization of the Asian regional fluxes influences the fluxes in the rest of the world. The inversion performance is examined by comparing the CO_2_ concentration from the optimized models against independent observations from the CONTRAIL programme between 2008 and 2010. This study focuses on the temporal evolution of the fluxes and the quantification of trends and their uncertainty. This study is part of an effort examining the Asian greenhouse gas budget initiated by RECCAP (REgional Carbon Cycle Assessment and Processes^20^) as part of the international Global Carbon Project (www.globalcarbonproject.org/reccap/).

## Results

### Uncertainties from fossil fuel and cement inventories

Atmospheric inversions optimize the total land/ocean to atmosphere CO_2_ flux (that is, the net biosphere plus the fossil fuel and cement (FFC) flux), therefore, the FFC fluxes must be subtracted *a posteriori* to obtain the land biosphere fluxes. However, the uncertainty in the FFC fluxes is not negligible. Although globally, the FFC flux is quite consistent among all inversions and with the CDIAC (Carbon Dioxide Information Analysis Center, 2013; http://cdiac.ornl.gov) inventory (within ±2%), this is not the case in Asia (Supplementary Fig. 1). In East Asia, the modelling groups assumed different mean values and trends in their FFC fluxes leading to disparities of up to ∼1 PgC per year (33%) in 2012, while in South and Southeast Asia, the absolute disparities were smaller but still significant, ∼0.2 PgC per year (33–50%) in 2012. The disparities arise from the different inventories on which their estimates were based, for example, CDIAC or EDGAR (Emission Database for Global Atmospheric Research, version 4.2; http://edgar.jrc.ec.europa.eu) and whether a static or evolving spatial distribution was used (see Supplementary Note 1).

Owing to the differences in prior FFC fluxes between inverse systems, it is recommended practice to subtract a standard FFC flux estimate from the total posterior fluxes when comparing land biosphere fluxes from different CO_2_ inversions^21^. However, since the total posterior flux may still be biased, and depends to a certain degree on the prior information, biases in the prior FFC fluxes may be propagated into the posterior land biosphere fluxes. In this case, the bias in the land biosphere flux will be proportional to the difference between the prior FFC flux used in each inversion, and the standard FFC flux (see Supplementary Fig. 2). An additional source of uncertainty comes from the choice of standard FFC flux that is subtracted; an overestimate of the standard FFC flux would lead to an overestimate of the land biosphere sink and vice versa. Commonly used FFC fluxes from inventories, i.e., CDIAC, EDGAR and IEA (International Energy Agency, 2014 edition; http://www.iea.org), differ substantially in the trend for East Asia from circa 2002 resulting in differences of ∼0.5 PgC per year (∼17%) in 2012, much higher than the uncertainty of the inventories of 8% (2σ) reported in ref. 22. (Note that IEA inventory does not include cement emissions so these were accounted for by using the cement emission estimates from CDIAC). Large inconsistencies have been previously reported in Chinese energy-use statistics and emission factors, leading to differences between the reported emissions of 14 to 18% in recent years^23^,^24^, which highlights the considerable systematic uncertainty in the FFC fluxes, especially for East Asia.

**Figure 1:**
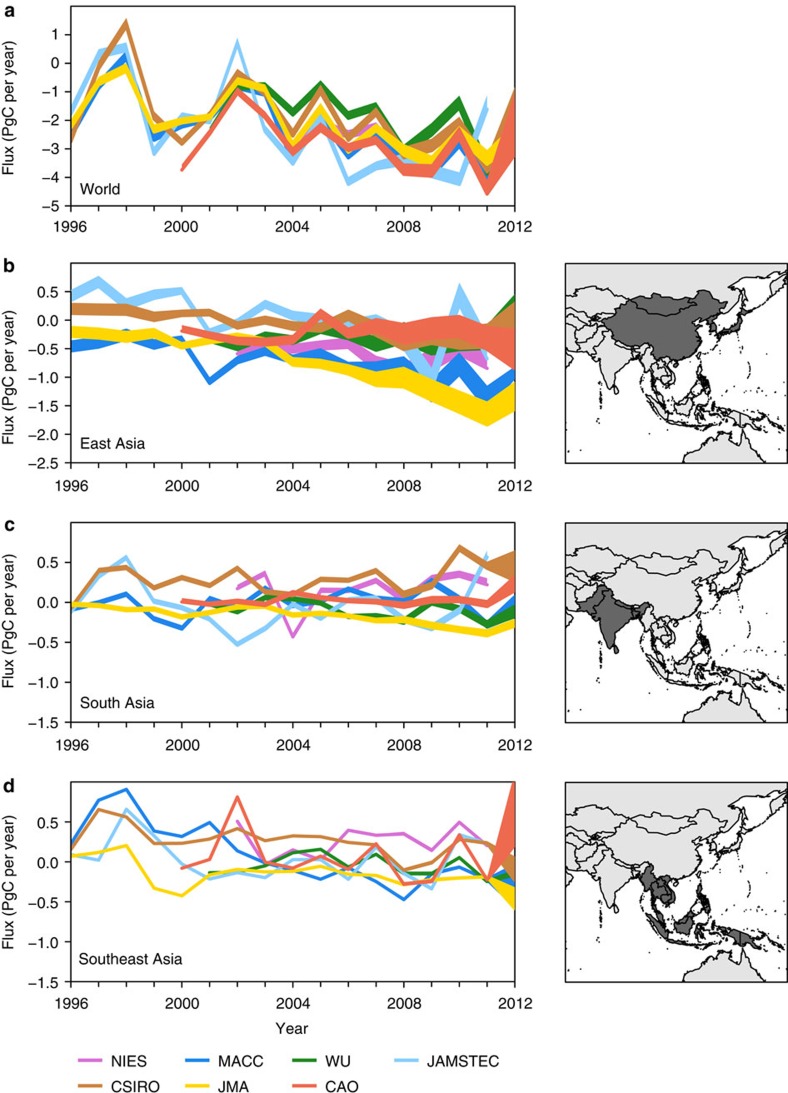
Land biosphere fluxes estimated by the atmospheric CO_2_ inversions. The posterior land biosphere (natural, land-use change and fire) fluxes (PgC per year) from each inversion. The width of each curve shows the range obtained by subtracting the FFC emissions estimates of the CDIAC, EDGAR and IEA inventories from the total posterior flux. Note that a negative flux indicates a carbon sink. The fluxes are shown globally (**a**) and for the regions: East Asia (**b**), South Asia (**c**) and Southeast Asia (**d**) as indicated in the adjacent maps. The inversions are named after the institute that performed them (NIES is the GELCA 64-region inversion system, MACC is the MACC-v13 inversion system used at LSCE, WU is CarbonTracker Europe, JAMSTEC is the ACTM-based inversion system, CSIRO is the CCAM-based inversion system, JMA is the JMA-CDTM-based inversion system, and CAO is the GELCA EOF-based inversion system, for details see Supplementary Table 1).

Figure 1 shows the posterior (optimized using atmospheric CO_2_ observations) land biosphere CO_2_ fluxes from each of the inversions (see also Table 1). Note that the land biosphere fluxes include natural, land-use change, and fire fluxes. We show the range of posterior land biosphere fluxes for each inversion calculated using three standard FFC fluxes from the most commonly used inventories: CDIAC, EDGAR and IEA (see Supplementary Note 2). The uncertainty in the posterior land biosphere fluxes increases over time, especially in East Asia, owing to disparities in the FFC fluxes. For the analyses in the following sections, we use an ensemble of seven inversions with three different standard FFC fluxes.

### Global land biosphere and ocean fluxes

Before discussing the Asian land biosphere fluxes, we first consider the global total as this provides a baseline for evaluating the inversion performance. The global posterior land fluxes from all inversions exhibit a high degree of consistency in the inter-annual variability and long-term trend (Fig. 1). All inversions show a reduction in the land sink (compared with the decadal mean) in 1997–1998 corresponding to a strong El Niño event (four inversions available) and in 2002–2003 corresponding to persistent El Niño conditions (7 inversions available). El Niño is associated with reduced precipitation, lower rates of net primary productivity (NPP), and increased fire disturbance in tropical and subtropical South America and Africa, Southeast Asia and Australia^25^,^26^,^27^. Six of the inversions also find an increased land sink in 2011, corresponding to a sustained La Niña period, which has been attributed to increased precipitation and net ecosystem uptake in semi-arid regions of South America, South Africa and Australia^28^. The 2011 land sink of −3.6±0.5 (−4.6 to −1.1) PgC per year (median±median absolute deviation, and full range of the ensemble) is the largest over 1996–2012 and is in good agreement with a recent process-based model estimate^28^ with a land sink of −3.9±1.3 PgC per year, which is greater than the 97.5th percentile over 1981–2012.

Over the period 1996–2007, we find a global land sink of −1.7±0.3 (−2.5 to −1.2) PgC per year consistent with two other estimates, based on different methods, for the same time period. The first is from the Global Carbon Project (GCP) and is calculated as the residual between the FFC emissions and the change in atmospheric CO_2_ plus the ocean flux based on ocean data^29^. GCP estimates a land sink (that is, the balance of land-use change and natural fluxes) of −1.00±0.64 PgC per year. This, however, does not include the riverine ‘loop' of the natural carbon cycle that existed before human perturbation and which is included in the inversion estimates. The riverine loop consists of a land biosphere uptake of CO_2_ of 0.45 PgC per year, which is transported via rivers to the ocean where it is outgassed to the atmosphere^30^. Adding the land biosphere flux associated with the riverine loop to the GCP estimate gives a land sink of −1.45±0.64 PgC per year, which is comparable to our estimate. The second estimate of the global land sink, based on the changes in atmospheric O_2_ and CO_2_ concentrations^31^, is −1.1±0.8 PgC per year, and again is not significantly different from our estimate owing to the large uncertainty. Furthermore, we find that the annual land biosphere sink increased by 1.7±0.2 (1.4–2.0) PgC between 1996–2001 and 2008–2012 (based on the four inversions with data before 2000). In most inversions, the sink increased in tropical South America, eastern North America, Siberia and East Asia and, in three inversions, also in Europe. A trend of increasing land sink over the past circa 15 years has also been identified in previous studies^32^,^33^. At least part of the observed trend may be attributable to the change in occurrence frequency of El Niño and La Niña events between 1996–2004 and 2005–2012: in the second period there were less frequent and intense El Niño events and a sustained La Niña event. To ascertain whether the land sink is increasing also in response to land management changes, CO_2_ and nitrogen fertilization, one would need a longer time series to filter-out climate oscillation effects, such as ENSO, and the influence of volcanic aerosols^34^. Process-based ecosystem models and forest inventories suggest that CO_2_ fertilization, nitrogen deposition and forest regrowth are largely driving a long-term increase in the land biosphere sink^33^.

The mean global ocean sink over 1996–2012 from all inversions is −1.6±0.3 (−2.1 to −1.2) PgC per year and is not statistically different from the estimate from a recent synthesis study^35^ for 1990–2009 of −1.55±0.6 PgC per year (note this includes a correction of 0.45 PgC per year for the riverine transport of carbon from the land to the ocean^30^, where it is eventually released to the atmosphere). We also find a small trend of increasing ocean sink (Mann Kendall test *P* value <0.1) with a mean annual increase of 0.01 PgC per year and is within the range of trends found by the synthesis study^35^ for the past two decades of 0.01–0.05 PgC per year. However, the ocean sink is somewhat dependent on the prior ocean flux estimates used in the inversions, and thus the two estimates are not completely independent.

### Assessment of the Asian fluxes

While the global fluxes from inversions are mass conserving, this is not the case on regional scales. Therefore, we evaluate the total posterior flux from each inversion by comparing atmospheric concentrations from forward simulations with independent observations (that is, not assimilated in the inversion) from CONTRAIL aircraft measurements^14^. The CONTRAIL measurements were made using an *in situ* analyser onboard commercial aircraft with regular flights from Japan to other Asian countries (for further details see Supplementary Note 3). We selected CONTRAIL data at seven locations where vertical profiles are available, that is, above the international airports at Bangkok, Singapore, Jakarta and Manila (used to evaluate the Southeast Asian fluxes), Seoul and Taipei (East Asian) and Delhi (South Asian) for 2008–2010. The models were sampled at the times and locations of the observations (for JMA and CSIRO only monthly mean model values were available).

**Figure 2:**
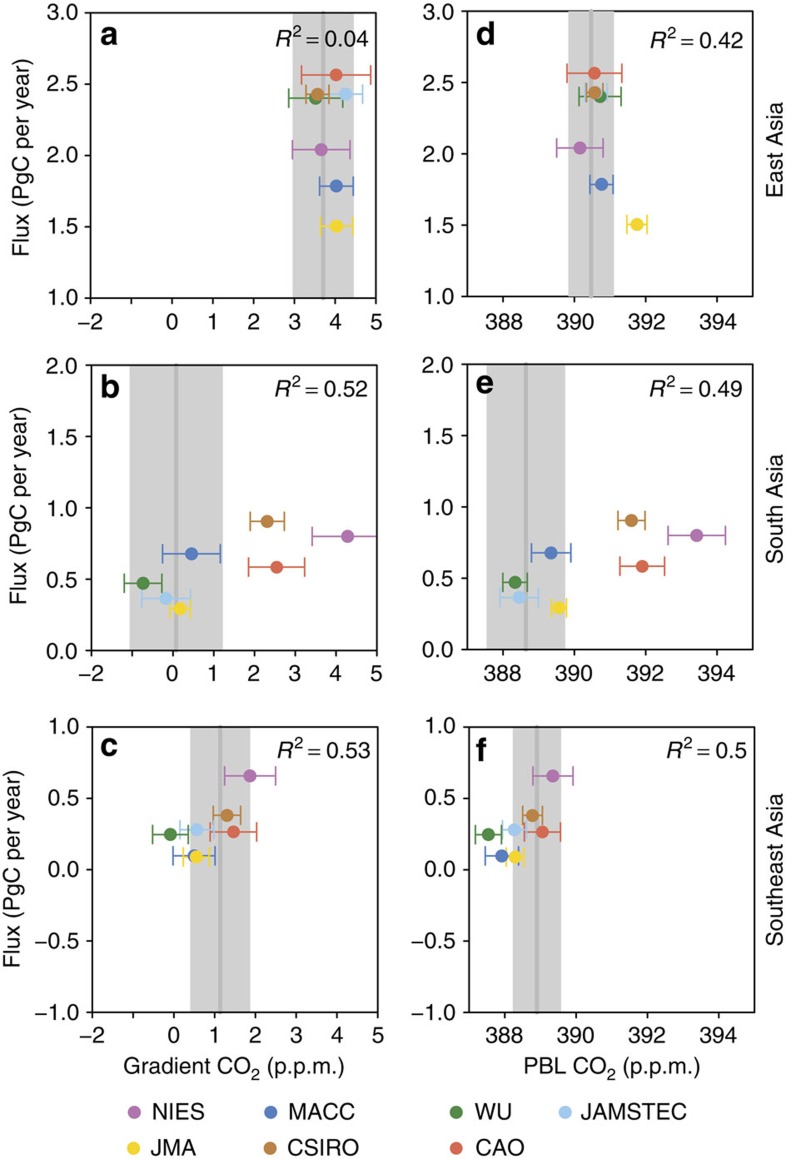
Posterior total fluxes versus CO_2_ vertical gradients and PBL concentrations for 2008–2010. The annual mean flux (PgC per year) versus CO_2_ gradient (p.p.m.) between 1 and 4 km for each inversion is shown for East Asia (**a**), South Asia (**b**) and Southeast Asia (**c**). Also shown is the annual mean flux versus the PBL CO_2_ concentration (p.p.m.) for East Asia (**d**), South Asia (**e**) and Southeast Asia (**f**). Each of the inversions was sampled at the times and locations of the CONTRAIL aircraft data and the error bars show the 1-*σ* s.d. of the inversion results. The grey vertical lines indicate the values of the observed gradients and PBL concentrations and the shading shows the 1-σ s.d. The correlation (*R*^2^) between the fluxes and the gradients and PBL concentrations is given in each plot.

To determine the importance of systematic errors in the inverse models for the regional flux estimates, we compared the annual mean posterior flux with the modelled and observed afternoon CO_2_ vertical gradients and planetary boundary layer (PBL) concentrations (Fig. 2). The vertical gradient was calculated between 1 and 4 km, that is, from the PBL to the free troposphere. As well as the annual mean, the comparison was made for winter (December to February, DJF) and summer (June to August, JJA) vertical gradients and PBL concentrations (Supplementary Fig. 3). Systematic over-/underestimation of the CO_2_ vertical gradient compared with that observed can indicate a bias in the posterior fluxes and may be due to too weak/strong vertical transport^36^.

For South and Southeast Asia, the annual vertical CO_2_ gradient and PBL concentration differences are positively correlated with the posterior fluxes, such that large modelled gradients and PBL concentrations correspond to large fluxes, and vice versa. This suggests that the model-observation differences are related to errors in the fluxes rather than to systematic biases in the vertical mixing between the PBL and free troposphere. In summer and winter, the model-observation differences for South Asia are much larger than for the other regions and may owe to the fact that the comparison is based on only one site, Delhi, which is continental and strongly influenced by local fluxes. For East Asia, there is no correlation between the annual (and winter) vertical gradients and the posterior fluxes, and the modelled gradients and PBL concentrations are close to those observed, suggesting that there is no significant bias in the annual mean (and winter) fluxes. In summer, however, the correlation is negative and the s.d. of the modelled and observed gradients is larger. This may result from the stronger influence from the southeasterly direction during this season, and hence the vertical gradients and PBL concentrations represent a combination of land and ocean influences.

Since the summer and winter model-observation differences are generally in the same direction for each model, that is, the summer and winter errors do not compensate, we use the annual mean model-observation differences to evaluate the posterior fluxes from each inversion. The annual means also contain the largest number of data points and are, therefore, more robust. For East and Southeast Asia, we do not find any strong bias in the gradients or PBL concentrations. Therefore, on this basis we do not omit any of the inversions from the ensemble for these two regions. However, for South Asia, the inversions NIES, CSIRO and CAO show large positive biases for the vertical gradient and PBL concentrations (>2 p.p.m.), therefore, we have omitted these results from the ensemble for this region.

### East Asia

We find that the East Asian land biosphere was on average a sink of −0.29 (−0.81 to 0.18) PgC per year for 1996–2012. Some of the differences among the inversion results for East Asia can be related to differences in the prior information (Supplementary Figs 1, 2 and 4). The JMA inversion used FFC emission estimates with a fixed spatial distribution based on that in 1995 and only scaled the global total. Because the growth rate in East Asia for the 2000s was higher than the global mean, this resulted in lower FFC and total fluxes in the JMA inversion for East Asia; the total posterior flux in JMA for 2008–2012 was 36% lower than the mean of the other inversions. Thus, subtracting the standard FFC flux from the JMA posterior total results in a more negative land biosphere flux compared with the other inversions. As CONTRAIL data were not available for the latter part of this period, when the bias is largest, this could not be seen in the comparison with independent atmospheric observations. In any case, to avoid potentially biasing the results, we exclude the JMA inversion from the ensemble statistics for East Asia for 2008–2012.

**Figure 3:**
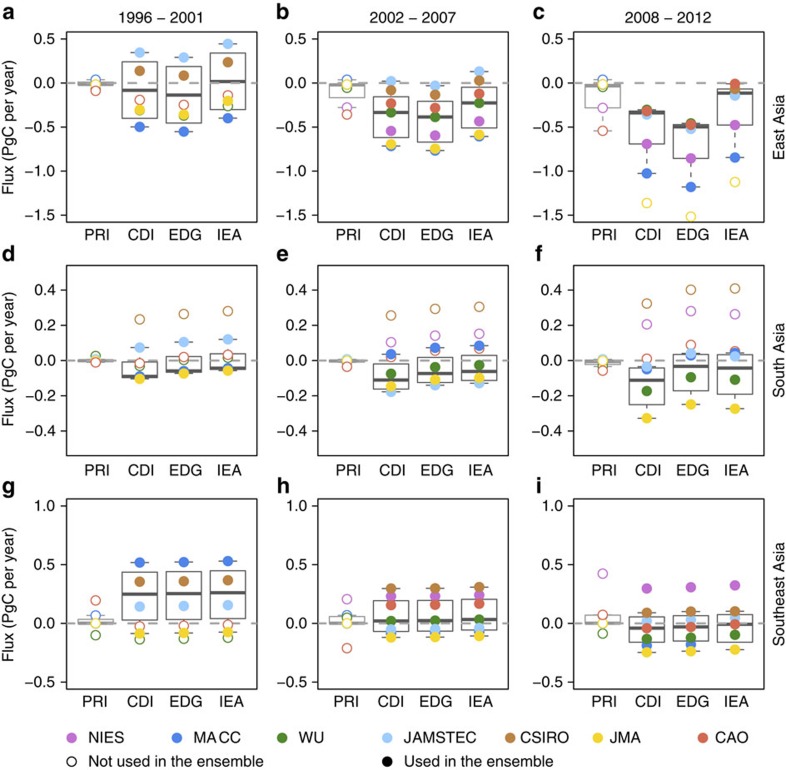
Summary of the land biosphere fluxes based on the inversion ensemble. Prior and posterior land biosphere fluxes (PgC per year) for intervals 1996–2001 (**a**,**d**,**g**), 2002–2007 (**b**,**e**,**h**) and 2008–2012 (**c**,**f**,**i**). The land biosphere fluxes are calculated by subtracting CDIAC (CDI), EDGAR (EDG) and IEA FFC emission estimates, respectively, from the total posterior fluxes. The thick horizontal bar within the box shows the median, the box extent shows the upper and lower quartiles, and the whiskers extend to the furthest point within the range of the median plus/minus the interquartile range. The open circles indicate the prior (PRI) and the solid circles the posterior estimate from the individual inversions. For East Asia, JMA was excluded for 2008–2012, while for South Asia, NIES, CSIRO and CAO were excluded from the ensembles and are also shown as open circles. Inversions that only started in 2000 or 2001 were also excluded from the 1996–2001 ensembles. The grey dashed line indicates the level of a neutral land biosphere (that is, zero flux).

The three inversions with data before 2001 (and excluding JMA), all indicate an increasing land sink (that is, the flux became more negative) over time (Mann Kendall test *P* value <0.01). From the ensemble, we estimate that the land sink increased from −0.06 (−0.55 to 0.44) PgC per year in 1996–2001 to −0.46 (−1.18 to −0.01) PgC per year in 2008–2012; with a median difference of 0.56 (0.30–0.81) PgC per year (Fig. 3). The three inversions (MACC, JAMSTEC and CSIRO) also show a small increase in the amplitude of the seasonal cycle in the East Asian land biosphere flux in 2008–2012 relative to 1996–2001 (Supplementary Fig. 5), while there was no trend in the amplitude of the seasonal cycle in the FFC fluxes used in the inversions.

**Figure 4:**
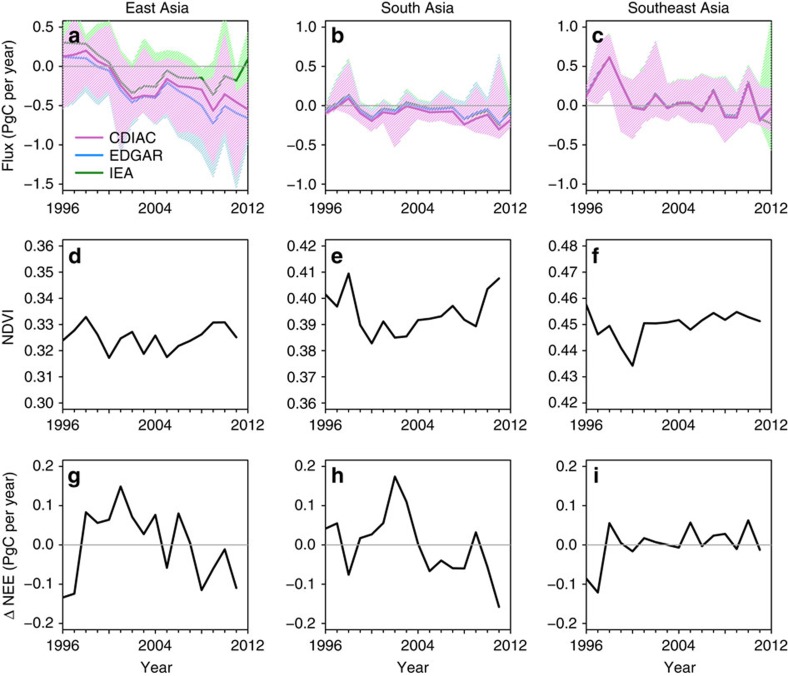
Trends in the posterior land biosphere fluxes. The land biosphere fluxes (PgC per year), NDVI and eddy-covariance-based NEE anomalies (PgC per year) are shown as annual means for East Asia (**a**,**d**,**g**), South Asia (**b**,**e**,**h**) and Southeast Asia (**c**,**f**,**i**). The fluxes are calculated using FFC emission estimates from CDIAC (purple), EDGAR (blue) and IEA (green), the solid line shows the median and the shading indicates the range of the inversion ensemble. For East Asia, JMA was excluded, while for South Asia, NIES, CSIRO and CAO were excluded. (Negative NEE indicates a carbon sink.)

Our land sink estimate for 2001–2005, of −0.33 (−0.77 to 0.10) PgC per year, is commensurate with a study using an ecosystem model^37^, which found an average sink for China of approximately −0.27 PgC per year for the same time period. Our results are also consistent with a recent study^38^ based on a combination of satellite measurements of greenness, process-based ecosystem models, and atmospheric inversions, which found a sink for East Asia of −0.22 to −0.29 PgC per year for 1990–2009, compared with our estimate of −0.25 (−0.74 to 0.18) PgC per year for 1996–2009. The increase in the land sink is supported by forest inventory data, which show an increase in the forest sink in China of ∼34% between 1990–1999 and 2000–2007 owing to intensive national afforestation/reforestation programmes^8^. To investigate this further, we examine the Normalized Difference Vegetation Index (NDVI; from the Advanced Very High Resolution Radiometer from NOAA satellites, see Supplementary Note 4) and an independent estimate of Net Ecosystem Exchange (NEE) based on an up-scaling model of CO_2_ eddy covariance data^39^. Figure 4 shows the median and range of the East Asian land biosphere flux given by the six inversions (excluding JMA) for the three standard FFC fluxes, as well as NDVI and the eddy-covariance-based NEE anomaly over East Asia. Although NDVI showed no significant trend, NEE became more negative (that is, a larger sink) over the same period by ∼0.2 PgC. Thus, a sink increase is supported by independent data and, in addition to afforestation/reforestation, may be driven by regional climate change; the temperature in East Asia increased by 0.4 °C between 1999 and 2009 (ref. 38).

The increase in the East Asian sink from the inversion ensemble is larger than suggested by either the eddy-covariance-based NEE or the forest inventory estimates. The larger sink increase in our estimates may be partly due to the uncertainty in the FFC fluxes. For example, using FFC fluxes with a low growth rate, such as IEA, the sink increase between 1996–2001 and 2008–2012 is 0.45 (0.30–0.59) PgC, while using a high growth rate estimate, such as EDGAR, it is 0.63 (0.56–0.81) PgC (Fig. 4). In summary, while atmospheric observations support an increase in the land biosphere sink in East Asia, the amount by which it has increased cannot be accurately determined until the uncertainty in FFC emissions is substantially reduced.

### South Asia

From the inversion ensemble, we find that the land biosphere in South Asia was close to carbon neutral, −0.05 (−0.18 to 0.03) PgC per year for 1996–2012. Similarly to East Asia, some differences between the inversion results are apparent owing to the FFC flux estimates. The JMA inversion used an FFC flux with a low growth rate (lower than the other inversions and the three standard FFC fluxes) and correspondingly this inversion shows the largest increase in land biosphere sink. On the other hand, the CSIRO inversion used a high FFC flux and this inversion also shows the most positive land biosphere flux.

While we find that the South Asian land biosphere was nearly carbon neutral, previous inversion studies^16^,^17^,^18^ found it to be a carbon sink of −0.10 to −0.37 PgC per year between 2002 and 2008. However, for the overlapping period of these studies, that is, 2007–2008, our estimate of −0.13 (−0.24–0.07) PgC per year is not significantly different. We also compare our results with a study based on a synthesis of 10 ecosystem models^40^ for the period 2000–2009. We find a smaller sink of −0.11 (−0.19 to 0.06) PgC per year compared with the ecosystem model estimate of −0.19±0.19 PgC per year.

Of the inversions with data before 2001, only one (JMA) show a significant trend in the land sink for South Asia and this may be due to the low growth rate in its FFC fluxes (Supplementary Fig. 2). Furthermore, we found no change in the amplitude of the seasonal cycle in land biosphere flux over the 17-year period, consistent with the findings of ecosystem models^40^. The overall absence of a trend is in contrast to the decrease in the eddy-covariance-based NEE estimate since 2002 (Fig. 4). Aside from possible errors in the eddy-covariance-based NEE (the discussion of which is outside the scope of our study) a possible explanation for the discrepancy is the poor atmospheric observation coverage for South Asia, and thus modest uncertainty reduction, 27% (9–37%, Supplementary Fig. 6), which may mean that the trend could not be detected. Alternatively, if the trend in FFC emissions were underestimated, this would also lead to an underestimation of the increase in the land sink from the inversions.

### Southeast Asia

For 1996–2001, three of the four inversions with data throughout this time period found the Southeast Asian land biosphere to be a source, and the ensemble median and range was 0.26 (−0.09 to 0.53) PgC per year. This period included the strong El Niño event of 1997–1998, which was associated with drought and lower rates of NPP, as well as increased fire disturbance^27^,^41^. For 2002–2012, however, we could not determine any difference from carbon neutrality for the land biosphere, −0.02 (−0.18 to 0.27) PgC per year. Two inversions (MACC and CSIRO) show a significantly increasing land sink in Southeast Asia over 1996–2012 (Mann Kendall test *P* value < 0.1), which mostly results from the large positive land biosphere flux (source) in 1997–1998 related to strong El Niño conditions. Only one inversion (NIES) estimates a significant source for 2002–2012. This can be explained by the use of a positive prior land flux, the influence of which can be seen in the posterior land fluxes.

Previous inversion studies have given contradicting results for Southeast Asia, that is, some estimate a source^17^ and others a sink^18^. For the average over 1996–2012, we find a weak source for the Southeast Asian land biosphere of 0.06 (−0.15 to 0.28) PgC per year, which is consistent with observed land-use changes (13% of forested area was lost in Southeast Asia between 1990 and 2010 (ref. 6)) and with estimates of above ground biomass loss of 0.09 PgC per year for the average over 1993–2012 based on satellite observations^42^. Our results, however, suggest that the Southeast Asian source reduced in 2002–2012 compared with 1996–2001, and all of the inversions with the data before 2001, show lower mean fluxes over the latter period. Furthermore, since 2001 the eddy-covariance-based NEE has been close to zero, while NDVI has been stable, supporting our finding of a near-neutral land biosphere for 2002–2012. The change to a neutral land biosphere may reflect the slowing rate of deforestation in Southeast Asia; according to FAO statistics the rate of deforestation slowed to 1.1 Mha per year between 2000 and 2010 compared with 2.7 Mha per year between 1990 and 2000 (ref. 6).

## Discussion

We have used an ensemble of seven atmospheric CO_2_ inversions and three global FFC flux estimates, based on the inventories of CDIAC, EDGAR and IEA, to infer the land biosphere fluxes for East, South and Southeast Asia, and to ascertain the robustness and overall uncertainty of the results. Asia as a whole emitted an average of 2.7–2.9 PgC per year over 1996–2012 from fossil fuel combustion and cement production with an average increase of ∼5% per year. Of this, an average of 17% (0–26%) of the emitted carbon was sequestered by the land biosphere, with most of the sink located in East Asia (Supplementary Fig. 7). This is smaller than the mean fraction, 28% (20–38%) of global fossil fuel emissions sequestered by the global land biosphere.

The East Asian land biosphere was on average a carbon sink of −0.29 (−0.81 to 0.18) PgC per year or equivalently 14% (0–39%) of East Asia's total FFC emissions, over 1996–2012. Between 1996–2001 and 2008–2012, we find an increase in the annual sink of 0.56 (0.30–0.81) PgC, which explains 35% of the increase in the global land sink. However, the magnitude of this increase in the East Asian sink is contingent on the assumed increase in FFC emissions (the inventories differ by up to 17% for East Asia). The uncertainty in the growth rate of the FFC emissions in the inventories contributes 32% to the uncertainty in the land sink change; if China's emissions have grown commensurate with the low rate estimate of for example, IEA, then the land sink increase will be at the lower end of the range, and vice versa if they have followed the high rate estimate of for example, EDGAR. This highlights the need for better regional estimates of FFC emissions and trends for determining land biosphere fluxes from inverse systems. For South Asia, we find that on average the land biosphere was close to carbon neutral, −0.05 (−0.18 to 0.03) PgC per year over 1996–2012 and that there was no significant trend. During this time period, there was only a modest net increase, ∼2 Mha, in forested area as afforestation/reforestation in India was nearly counter balanced by deforestation in Nepal and Pakistan^6^. For Southeast Asia, we find that the land biosphere was on average a carbon source between 1996 and 2001 of 0.26 (−0.09 to 0.53) PgC per year but between 2002 and 2012, we cannot determine any difference from carbon neutrality with a flux of −0.02 (−0.18 to 0.27) PgC per year.

Our results contrast with the general findings of land surface models^33^, which allocate the increase in the global land sink to tropical and southern regions with little change to even a decrease in the temperate land sink over the past two decades. This disparity is likely owing to land-use changes; for instance, the regrowth of forests is not accounted for in land surface models but probably contributes substantially to the increasing land sink in East Asia^8^. While in tropical Asia the land biosphere models likely overestimate the increase in the sink, again by not accounting for land-use and land-cover changes, and because few land surface models include a coupled carbon–nitrogen cycle and as a consequence may have a too strong CO_2_ fertilization response.

## Methods

### Inversion methodology

All of the inversion systems used in this study have been previously described^16^,^21^,^43^,^44^,^45^,^46^,^47^,^48^ and are summarized in Supplementary Table 1. Atmospheric CO_2_ inversions optimize the land/ocean to atmosphere fluxes using spatiotemporal gradients in atmospheric CO_2_ concentration from observations. The relationship between the fluxes, **x** (the quantity to be optimized), and CO_2_ concentration, **y**, is described by an atmospheric transport model, *H*, and can be written as:


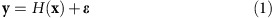


where ***ɛ*** is error associated with model representation (such as scale differences between the model and the observations), transport and measurements. Since there is insufficient information to solve equation (1) for the fluxes, statistical optimization methods are used, which require prior information. Using the Bayesian formalism, the problem can be expressed as the maximization of the probability density function of the fluxes given the prior information and observations and is equivalent to finding the minimum of the cost function:


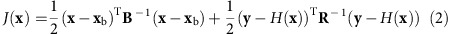


where **B** and **R** are the error covariance matrices for the prior fluxes and the observations, respectively. The fluxes that minimize the cost function can be found by solving the first order derivative of equation (2), and requires the gradient of the transport operator. The inversion systems can be grouped into three basic categories according to the way they minimize the cost function: (1) inversions using a variational method and an adjoint model of transport and which resolve the fluxes at the spatial resolution of the transport model (MACC); (2) inversions using a Bayesian synthesis for a number of predefined regions (JMA, JAMSTEC and CSIRO); and (3) inversions using Kalman smoother methods (WU, NIES and CAO). In each framework, the total CO_2_ flux is optimized and the posterior land biosphere fluxes are found by subtracting a standard FFC emissions estimate from the total. For access to the inversion and/or processing code, a request should be sent to the authors.

### Prior information and observations

For the prior land biosphere fluxes, JAMSTEC, CSIRO and JMA used estimates based on the ecosystem model, CASA^49^ while WU used the SiBCASA model^50^, MACC used the ORCHIDEE model^51^ and NIES and CAO used the VISIT model^52^. For the prior ocean fluxes, JAMSTEC, CSIRO, JMA and MACC used a pCO_2_ observation-based estimate^53^, WU used ocean fluxes estimated from an ocean-data inversion^30^, while NIES and CAO used fluxes from the OTTM ocean-data inversion model^54^. The observation dataset used also differed among the inversions (Supplementary Fig. 8). NIES, CAO and WU used observations from the NOAA ESRL GMD Observation Package^55^ and assimilated values at the time of sampling, or in the case on WU, as daily afternoon averages for sites below 1,000 m and daily morning averages for sites above 1,000 m. CSIRO and JAMSTEC used the GlobalView-CO_2_ package^56^ at monthly intervals. JMA also used monthly mean observations but used its own set of observations including 69 ground-based sites and 21 ocean-based sites as well as aircraft profiles and ship transects. MACC used 33 *in situ* sites and 106 flask sites and assimilated the data as daily afternoon averages for sites below 1,000 m and daily morning averages for sites above 1000 m.

## 

## Additional information

**How to cite this article:** Thompson, R. L. *et al*. Top–down assessment of the Asian carbon budget since the mid 1990s. *Nat. Commun.* 7:10724 doi: 10.1038/ncomms10724 (2016).

## Supplementary Material

Supplementary Information Supplementary Figures 1-8, Supplementary Table 1, Supplementary Notes 1-4 and Supplementary References.

## Figures and Tables

**Table 1:** Fossil fuel and cement emissions and land and ocean fluxes of CO_2_.

**Period**	**Air-borne fraction**	**Global FFC**	**Global Land**	**Global Ocean**	**East Asia FFC**	**East Asia Land**	**South Asia FFC**	**South Asia Land**	**Southeast Asia FFC**	**Southeast Asia Land**
1996–2001	0.52*0.54* (0.48–0.54)	6.69 (6.64–6.83)	−1.58 (−3.11 to −1.13)	−1.70 (−2.45 to −1.29)	1.48 (1.35–1.65)	−0.06 (−0.55 to 0.44)	0.35 (0.28–0.46)	−0.06 (−0.10 to 0.12)	0.22 (0.17–0.28)	0.26 (−0.09 to 0.53)
										
2002–2007	0.56 *0.57* (0.53–0.60)	7.86 (7.55–7.94)	−1.94 (−2.51 to −1.09)	−1.57 (−2.13 to −0.65)	2.03 (1.59–2.31)	−0.33 (−0.77 to 0.13)	0.45 (0.33–0.59)	−0.09 (−0.18 to 0.08)	0.27 (0.22–0.37)	0.02 (−0.12 to 0.31)
										
2008–2012	0.46*0.46* (0.44–0.49)	9.12 (8.87–9.24)	−3.17 (−3.65 to −2.28)	−1.77 (−2.36 to −1.31)	2.85 (1.85–3.01)	−0.46 (−1.18 to −0.01)	0.64 (0.39–0.83)	−0.07 (−0.33 to 0.04)	0.34 (0.26–0.45)	−0.03 (−0.25 to 0.32)

FFC, fossil fuel and cement.

The median (and range) of the FFC emissions, land biosphere and ocean flux estimates from the inversions (PgC per year) are given. Note that negative numbers indicate a flux from the atmosphere to the land/ocean. The land biosphere fluxes (natural, land-use change and fire) are from the inversions and using the CDIAC, EDGAR, and IEA FFC emission estimates. For East Asia, JMA was excluded for the period 2008–2012. For South Asia, NIES, CSIRO and CAO were excluded. Also shown is the median (and range) of the global air-borne fraction of the FFC emissions from the inversions and calculated directly from atmospheric observations (in italics; data from the Global Carbon Project).
