# Supplementary material for: Top–down assessment of the Asian carbon budget since the mid 1990s
Source: Nat Commun. 2016 Feb 25;7:10724. doi: 10.1038/ncomms10724 (PMC4773423; doi:10.1038/ncomms10724)
Supplement: Supplementary Information — Supplementary Figures 1-8, Supplementary Table 1, Supplementary Notes 1-4 and Supplementary References. [file ncomms10724-s1.pdf]

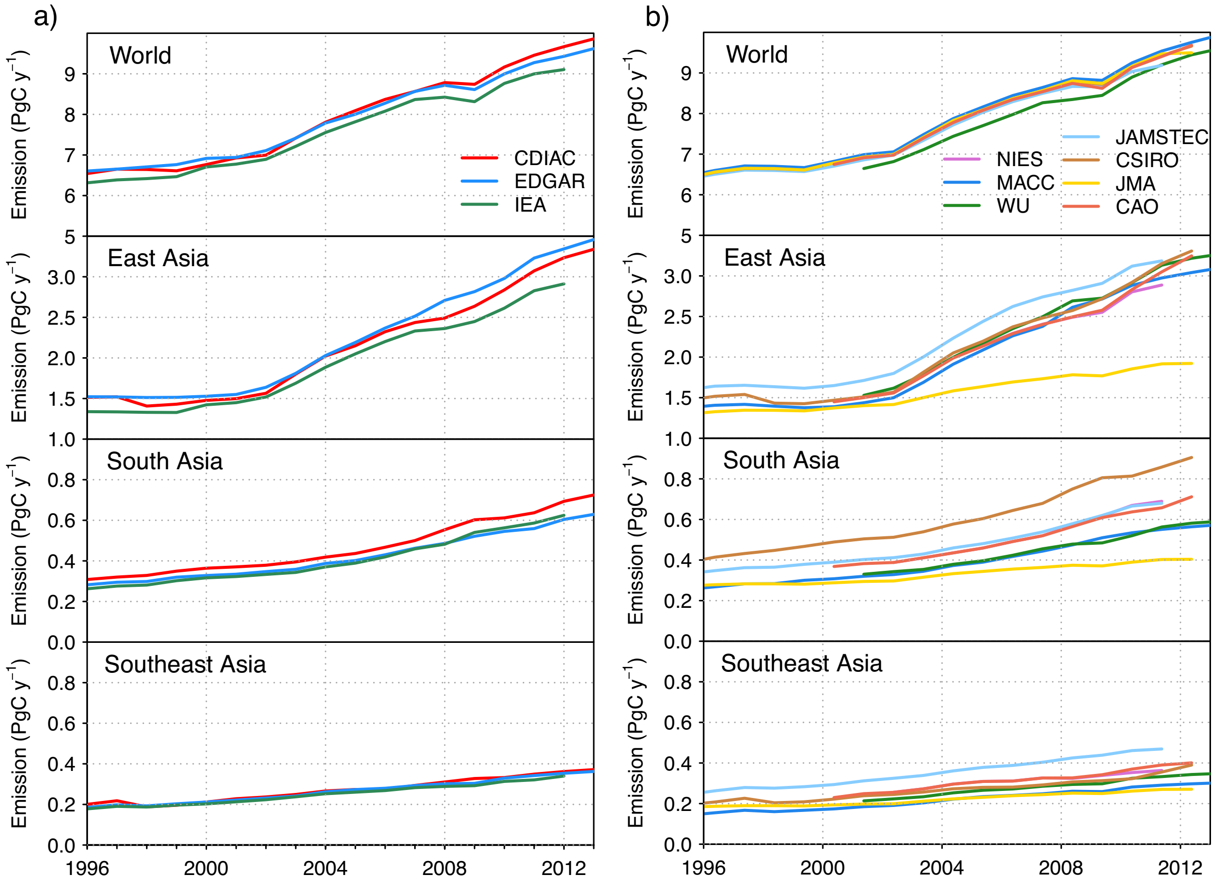

Supplementary Figure 1. Comparison of the annual FFC emissions (PgC y^{-1}) from CDIAC, IEA and EDGAR (a) and the annual FFC emissions used in each of the inversion systems (b).

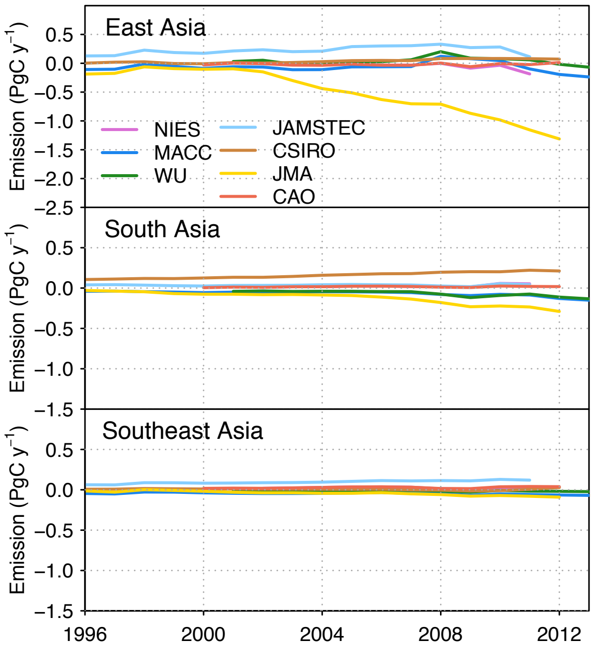

Supplementary Figure 2. Differences between the FFC emission estimates (PgC y^{-1}) used in each inversion and CDIAC for the regions of East Asia, South Asia and Southeast Asia. Since the global total in each inversion is scaled to be consistent with the CDIAC global total (except WU which used EDGAR) this difference is not significantly different from zero and, therefore, is not shown.

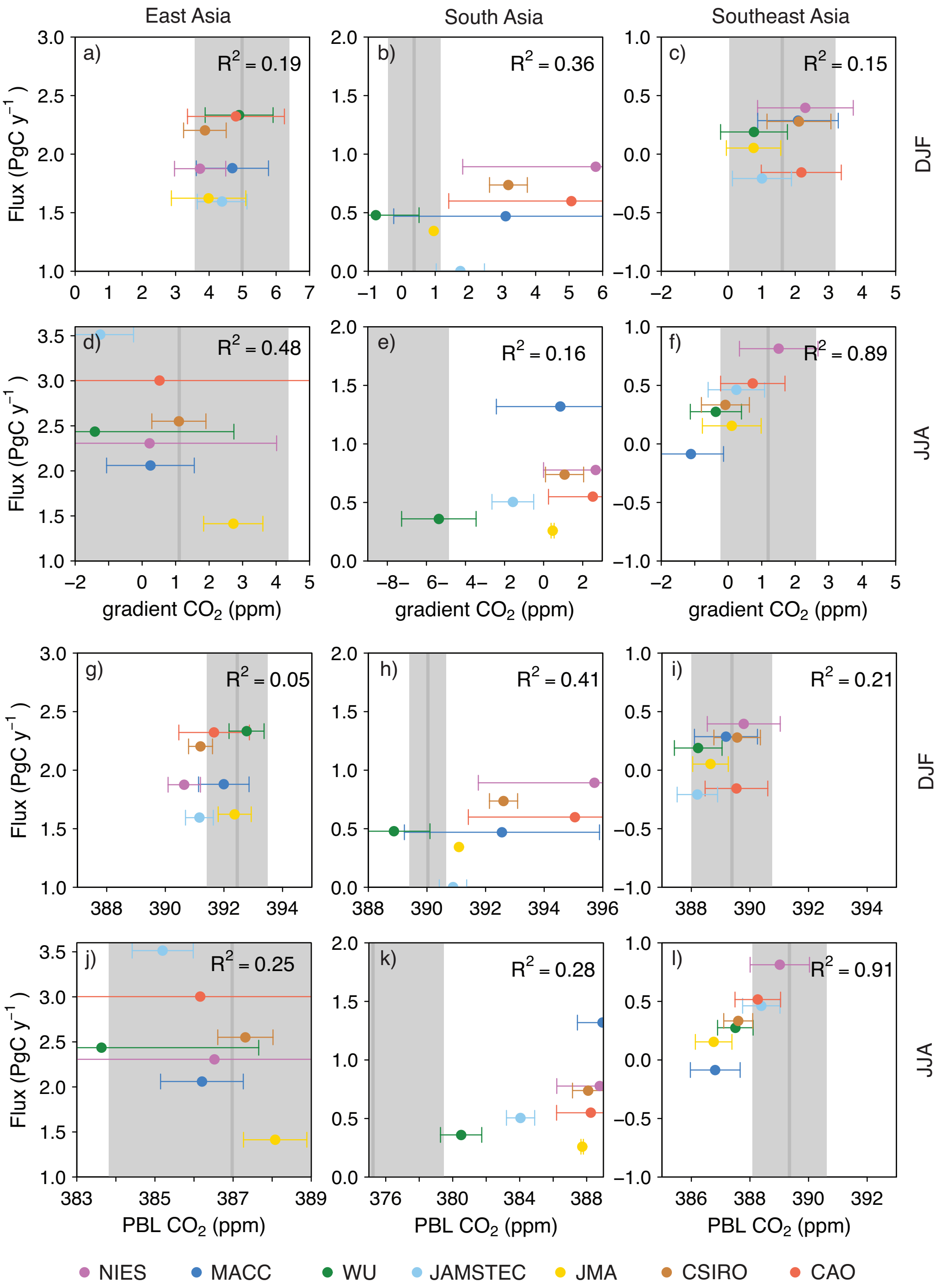

Supplementary Figure 3. Posterior total fluxes (PgC y^{-1}) versus CO_2 vertical gradients and PBL concentrations (ppm) for 2008 – 2010. The seasonal (December to February and June to August) fluxes versus CO_2 gradients between 1 and 4 km for each inversion is shown for East (a and d), South (b and e) and Southeast Asia (c and f). Also shown are the seasonal fluxes versus the PBL CO_2 concentrations for East (g and j), South (h and k) and Southeast Asia (i and l). Each of the inversions was sampled at the times and locations of the CONTRAIL aircraft data and the error bars show the 1-σ standard deviation of the inversion results. The gray line indicates the values of the observed vertical gradients and PBL concentrations and the shading shows the 1-σ standard deviation. The correlation (R^2) between the fluxes and the vertical gradients and PBL concentrations is given in each plot.

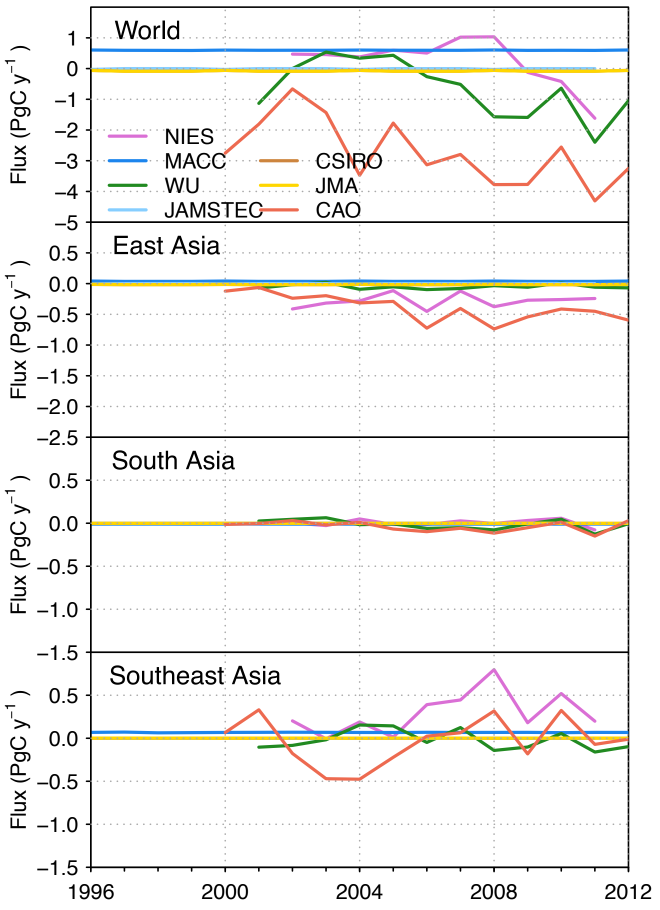

Supplementary Figure 4. Prior land biosphere flux estimates (Pg C y^{-1}) used in each inversion.

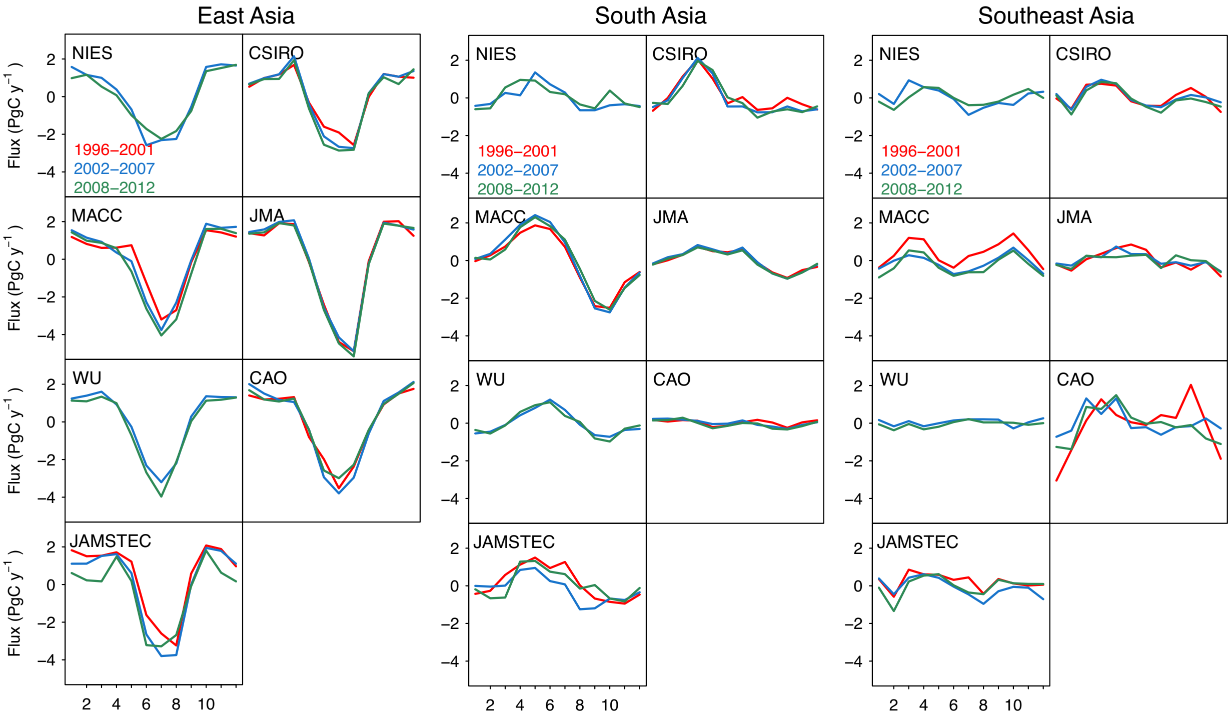

Supplementary Figure 5. Seasonal cycles of the posterior land biosphere fluxes (PgC y^{-1}). To avoid influences of different seasonal cycles in the FFC estimates, the prior FFC estimate used in each inversion was subtracted for the calculation of the posterior land biosphere flux seasonal cycles. The seasonal cycles were calculated as the monthly means of the detrended data (i.e. no curve fitting was applied).

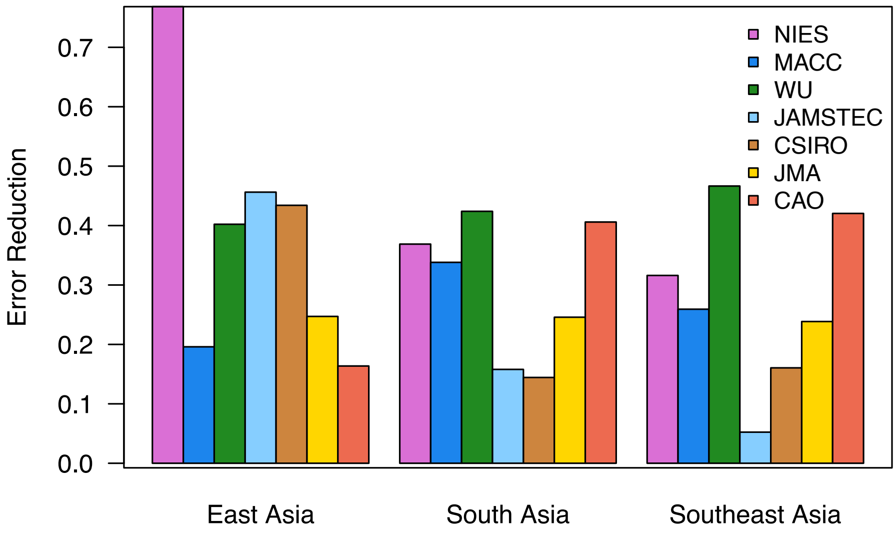

Supplementary Figure 6. Shown is the 2001 – 2012 mean fractional error reduction (i.e., one minus the ratio of the posterior to prior uncertainty).

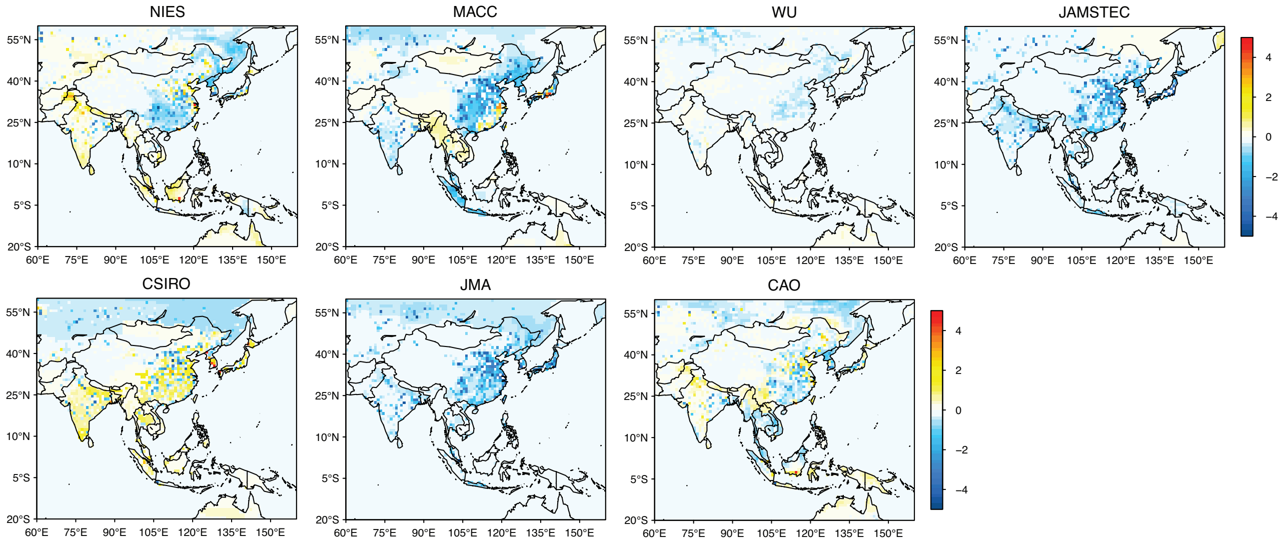

Supplementary Figure 7. Maps of mean annual posterior land biosphere fluxes ($kgCO_2$ m^{-2} y^{-1}) for 2001 to 2012 (overlapping period of all inversions). Positive fluxes are in the direction from the surface to the atmosphere. The land biosphere fluxes were calculated by subtracting a standard FFC flux (EDGAR v4.2) from the total posterior flux from each inversion.

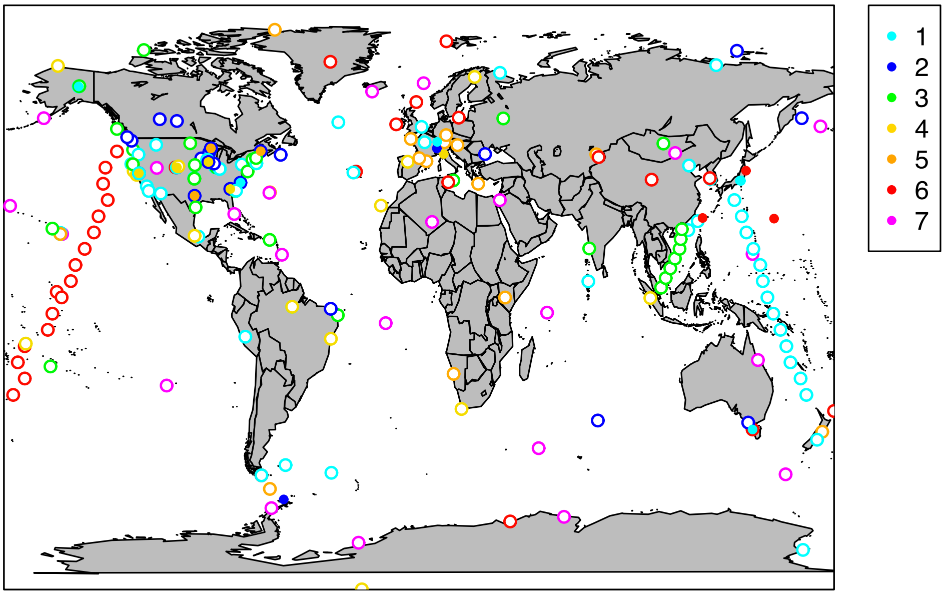

Supplementary Figure 8. Map of all sites used in the inversion ensemble. The color code represents the number of inversions that used each site and open/solid circles indicate whether the site is a flask/in-situ site, respectively. Note that some inversions additionally used ship transects and aircraft profiles, which are not shown on this map.

Supplementary Table 1. Overview of the inversion systems including information on the atmospheric transport model used and the compilation of the prior fossil fuel emissions estimates.

Transport model	Institute (ID)	Transport model resolution Horizontal	Vertical	Meteorology	State vector resolution	Fossil fuel emissions
GELCA[1]	NIES (NIES)	2.5° × 2.5° (1.25° × 1.25°)[a]	32 sigma-theta pressure layers	JCDAS	42 land, 22 ocean regions	ODIAC, scaled global total to CDIAC up to 2008, and BP statistics thereafter[8]
LMDZ[2]	LSCE (MACC[b])	3.75° × 1.875°	39 eta pressure layers	ECMWF	3.75° × 1.875°	EDGAR version 4.2, scaled global total to CDIAC
TM5[3]	WU (WU)	3.0° × 2.0° (zoom of 1.0° × 1.0° over Europe and N. America)	25 vertical pressure layers	ECMWF	1.0° × 1.0° over Europe and N. America, ecoregions rest of the world[c]	EDGAR version 4.2 with IER time profiles for specific countries and sectors[d]
ACTM[4]	JAMSTEC (JAMSTEC)	2.8° × 2.8°	32 sigma pressure layers up to 45 km	NCEP2	84 regions	EDGAR version 4.2, scaled global total to CDIAC
CCAM[5]	CSIRO (CSIRO)	208 × 208 km	18 sigma pressure layers up to 26 km	NCEP	146 regions	within region spatial distribution TransCom4, scaled regional totals to CDIAC
JMA_CDTM[6]	JMA/MRI (JMA)	2.5° × 2.5°	32 layers	JRA-25 (up to 2006) and JCDAS thereafter	22 regions as for TransCom3	TransCom3 fixed distribution, scaled global total to CDIAC
GELCA[1]	CAO (CAO)	2.5° × 2.5° (1.25° × 1.25°)[a]	32 sigma-theta pressure layers	JRA-25, JCDAS	coefficients of 327 empirical orthogonal functions[7]	ODIAC, scaled global total to CDIAC up to 2008, and BP statistics thereafter[8]

[a]coupled Lagrangian particle dispersion model, FLEXPART
[b]This is the MACC-v13.1 inversion, abbreviated as MACC in the text
[c]http://www.carbontracker.eu/documentation_assim.html#ct_doc
[d]http://www.carbones.eu/wcmqs/project/ccdas/#Fossil%20Fuel

Supplementary Note 1

This study includes seven independent inversion systems each with a different transport model, prior land biosphere and ocean fluxes, as well as fossil fuel and cement (FFC) emission estimates (see Supplementary Table 1). In all inversions, the prior FFC emissions estimate was scaled so that the annual global total was approximately consistent with the CDIAC inventory (http://cdiac.ornl.gov/trends/emis/meth_reg.html), except WU, which used the EDGAR version 4.2 inventory. However, the spatial distribution and temporal variability among inversions differed (Supplementary Table 1). Both JMA and CSIRO used a fixed spatial distribution (time invariant) for the fluxes. While CSIRO scaled the regional total emissions to match CDIAC, JMA scaled the global total emission, which led to much lower rates of FFC emission increase for East Asia after 2000 (since the East Asian emissions increased faster than the global mean). NIES and CAO used the ODIAC inventory[8], while the remaining inversions based the spatial distribution on EDGAR version 4.2 (http://edgar.jrc.ec.europa.eu). (The prior FFC emissions for each inversion system are shown in Supplementary Fig. 1 and 2).

Supplementary Note 2

In the post processing and analysis, we used FFC emission estimates from three different inventories, CDIAC (http://cdiac.ornl.gov/trends/emis/meth_reg.html), IEA (https://www.iea.org) and EDGAR version 4.2 (http://edgar.jrc.ec.europa.eu). The posterior land biosphere fluxes were calculated by subtracting each of these FFC estimates from the total posterior fluxes. In each inventory, the bunker fuels, i.e., fuel used in international aviation and maritime transport, were included in the global total but not in the regional totals. The IEA inventory does not include estimates for emissions from cement production, therefore, the estimates for this source from CDIAC were added to the IEA estimates.

Supplementary Note 3

The NOAA ESRL GMD Observation Package[9] brings together direct (ground-based) measurements of CO_2 from multiple laboratories in a standard format along with detailed meta-data. Details about the Observation Package and a link to download the data is available here: http://www.esrl.noaa.gov/gmd/ccgg/obspack/. The GlobalView-CO_2 package[10] is a data product based on a selection of concentration data for baseline conditions at each observation site. Details about GlobalView-CO_2 and a link to download the data is available here: http://www.esrl.noaa.gov/gmd/ccgg/globalview/co2/co2_intro.html.

For validation, we have used the CONTRAIL (Comprehensive Observation Network for TRace gases by AIrLiner) data[11]. The CONTRAIL measurements are made from commercial aircraft flying on a regular basis between Japan East, South and Southeast Asia, as well as to Australia, Hawaii and North America. We have used measurements made using an in-situ analyzer, i.e., the CONTRAIL Continuous CO_2 Measuring Equipment (CME). The CME consists of a LICOR LI-840 non-dispersive infrared analyzer, data logger and gas handling instrumentation (for details see reference 4). Air is sampled from an inlet mounted at the aircraft's air conditioning intake duct. Details about CONTRAIL and how to access the data can be found here: http://www.cger.nies.go.jp/contrail/index.html.

Supplementary Note 4

Vegetation activity data were analysed with Advanced Very High Radiometric Resolution (AVHRR) observations of the Normalized Difference Vegetation Index (NDVI). NDVI

measures vegetation greenness as the normalized difference between red and infra-red spectral bands. The data used here are the GIMMS 3G product available from 1982-2012[12].

Supplementary References

1. Ganshin, A., Oda, T., Saito, M., Maksyutov, S., *et al.* A global coupled Eulerian-Lagrangian model and 1× 1 km CO_2 surface flux dataset for high-resolution atmospheric CO_2 transport simulations. *Geosci. Model Dev* **5,** 231-243 (2012).
2. Hourdin, F. & Talagrand, O. Eulerian backtracking of atmospheric tracers. I: Adjoint derivation and parametrization of subgrid-scale transport. *Q. J. Roy. Meteor. Soc.* **132,** 567-583 (2006).
3. Krol, M., Houweling, S., Bregman, B., van den Broek, M., *et al.* The two-way nested global chemistry-transport zoom model TM5: algorithm and applications. *Atmos. Chem. Phys.* **5,** 417-432 (2005).
4. Patra, P. K., Takigawa, M., Dutton, G. S., Uhse, K., *et al.* Transport mechanisms for synoptic, seasonal and interannual SF_6 variations and "age" of air in troposphere. *Atmos. Chem. Phys.* **9,** 1209-1225 (2009).
5. McGregor, J. L., & Dix, M. R., An updated description of the conformal-cubic atmospheric model, in *High resolution numerical modelling of the atmosphere and ocean*, 51-75, Springer (2008).
6. Sasaki, T., Maki, T., Oohashi, S. & Akagi, K., Optimal sampling network and availability of data acquired at inland sites, in *Global Atmosphere Watch Report No. 148,* 77-79 (2003).
7. Zhuravlev, R., Khattatov, B., Kiryushov, B. & Maksyutov, S. Technical Note: A novel approach to estimation of time-variable surface sources and sinks of carbon dioxide using empirical orthogonal functions and the Kalman filter. *Atmos. Chem. Phys.* **11,** 10305-10315 (2011).
8. Oda, T. & Maksyutov, S. A very high-resolution (1 km × 1 km) global fossil fuel CO_2 emission inventory derived using a point source database and satellite observations of nighttime lights. *Atmos. Chem. Phys.* **11,** 543-556 (2011).
9. Cooperative Global Atmospheric Data Integration Project, updated annually. Multi-laboratory compilation of atmospheric carbon dioxide data for the period 2000-2012 (obspack_co2_1_PROTOTYPE_v1.0.4_2013-11-25), NOAA Global Monitoring Division, Boulder, Colorado, U.S.A, doi:10.3334/OBSPACK/1001
10. Cooperative Global Atmospheric Data Integration Project. 2013, updated annually. Multi-laboratory compilation of synchronized and gap-filled atmospheric carbon dioxide records for the period 1979-2012 (obspack_co2_1_GLOBALVIEW-CO2_2013_v1.0.4_2013-12-23). Compiled by NOAA Global Monitoring Division: Boulder, Colorado, U.S.A., doi:10.3334/OBSPACK/1002
11. Machida, T., Matsueda, H., Sawa, Y., Nakagawa, Y., et al. Worldwide measurements of Atmospheric CO2 and Other Trace Gas Species Using Commercial Airlines. J. Atmos. Ocean. Tech. 25, 1744-1754 (2008).
12. Zhu, Z., Bi, J., Pan, Y., Ganguly, S., *et al.* Global data sets of vegetation leaf area index (LAI) 3g and Fraction of Photosynthetically Active Radiation (FPAR) 3g derived from Global Inventory Modeling and Mapping Studies (GIMMS) Normalized Difference Vegetation Index (NDVI3g) for the period 1981 to 2011. *Remote Sensing* **5,** 927-948 (2013).
